# Pyridoxine Deficiency and Neurologic Dysfunction: An Unlikely Association

**DOI:** 10.7759/cureus.47647

**Published:** 2023-10-25

**Authors:** John M Sousou, Emma M Griffith, Christopher Marsalisi, Pramod Reddy

**Affiliations:** 1 Internal Medicine, University of Florida College of Medicine – Jacksonville, Jacksonville, USA

**Keywords:** vitamin b6, chronic alcoholism, myoclonic jerks, seizures, pyridoxine

## Abstract

Pyridoxine deficiency is a prevalent condition in the United States that primarily affects patients with alcohol use disorder. The presentation of this condition is very nonspecific and commonly presents with a constellation of symptoms including peripheral neuropathy, stomatitis, dermatitis, confusion, depression, encephalopathy, and seizures. Over half of these patients have associated alcohol use disorder, which causes pyridoxine deficiency due to the breakdown of pyridoxal phosphate during ethanol metabolism in the liver. As an important cofactor in the synthesis of γ-aminobutyric acid (GABA), deficient levels of pyridoxine may lower the seizure threshold due to reduced GABA-mediated inhibition. This case details a 57-year-old male with chronic alcoholism and a history of seizures who developed episodes of myoclonic jerks, tremors, anxiety, and neuropathy whose symptoms persisted even while on anti-epileptic medication. He was found to have pyridoxine deficiency and had full resolution of symptoms shortly after the administration of vitamin B6 supplementation. Pyridoxine deficiency may lead to severe neurologic disorders such as encephalopathy and seizures. Hence, it is important to consider pyridoxine deficiency in the workup of neurologic complaints, especially in high-risk patients.

## Introduction

Vitamin B6 (pyridoxine) is a water-soluble vitamin whose active form, pyridoxal 5-phosphate (PLP), serves as a cofactor for over one hundred enzymes involved in gluconeogenesis, glycogenolysis, metabolism of amino acids, carbohydrates, and lipids as well as the synthesis of heme and neurotransmitters [1]. Vitamin B6 also functions in regulating the nervous and endocrine systems. Abundant levels of vitamin B6 can be found in almost all food groups including fruits, vegetables, meat, legumes, and fish such as salmon or tuna. Nearly 13% of adults in the United States are deficient in vitamin B6 [2]. Pyridoxine deficiency is a well-known adverse effect of certain medications, such as isoniazid, carbidopa-levodopa, hydralazine, penicillamine, cycloserine, and theophylline; thus, it is imperative for patients to supplement vitamin B6 while on these drugs. Pyridoxine deficiency is also associated with alcohol use due to reduced dietary intake as well as the breakdown of pyridoxal phosphate by increased levels of pyridoxal phosphate phosphatase during ethanol metabolism in the liver, which occurs in over 50% of patients with alcohol use disorder [3]. Additionally, end-stage renal disease is associated with pyridoxine deficiency through multiple mechanisms, including the consumption of erythrocyte vitamin B6 by hemoglobin synthesis during erythropoietin (EPO) treatment in hemodialysis patients [4].

Typical signs and symptoms of vitamin B6 deficiency include peripheral neuropathy, stomatitis, dermatitis, confusion, depression, and elevated homocysteine levels. Importantly, neurologic disorders such as encephalopathy, abnormal electroencephalogram (EEG) readings, and seizures have been demonstrated in both infants and, in rare instances, adults [5]. As an important cofactor in the synthesis of γ-aminobutyric acid (GABA), pyridoxine deficiency results in impaired production of GABA which lowers the seizure threshold due to reduced GABA-mediated inhibition [6].

We present a case detailing a 57-year-old male with chronic alcoholism and previous seizures one month prior to admission who developed episodes of myoclonic jerks, tremors, and neuropathy. His symptoms persisted even after continued treatment and dose adjustment of levetiracetam, an anti-epileptic medication; interestingly, he became completely asymptomatic shortly after administration of pyridoxine supplementation.

## Case presentation

The patient is a 57-year-old male with a past medical history of non-ischemic cardiomyopathy, heart failure with a reduced ejection fraction of 25-30%, end-stage renal disease on hemodialysis, hypertension, hyperlipidemia, cocaine use, seizure disorder, and chronic alcohol use disorder (last alcoholic beverage five days prior to admission). He initially presented to the emergency department due to chest pain. Ischemic workup was ultimately negative. His hospital course was complicated by COVID-19 infection with intermittent hypoxic respiratory failure and recurrent hyperkalemia despite dialysis sessions. Due to the patient's prolonged hospitalization of over fifty days, as well as protein malnutrition, he developed notable physical deconditioning for which he worked with physical therapy.

On day 37 of his hospitalization, the patient began to complain of worsening myoclonic jerks manifesting as rapid intermittent jerking movements in all four extremities, hand tremors, and episodic numbness and tingling in his upper and lower extremities. Of note, he had multiple reported seizures one month prior to this admission and had been on levetiracetam 500 mg twice a day. Due to these symptoms, the patient struggled with activities of daily living including eating, drinking, and ambulating. These symptoms continued to worsen over the next several days, especially after his dialysis sessions and the resolution of his refractory hyperkalemia following a recirculation study with nephrology. Daily workup was negative for electrolyte disturbances (such as hyponatremia, hypernatremia, hypocalcemia, hypomagnesemia, and hypophosphatemia), infectious process, or causative medication. After consulting with inpatient Neurology and Nephrology, there was concern for the potential development of seizures with his worsening continuous myoclonic jerks and previous history of seizures one month prior. The patient’s dose of levetiracetam was adjusted by adding an additional 500 mg supplement dose immediately after his dialysis sessions three times per week. At the time, an EEG was deferred by Neurology due to the patient's symptoms being consistent with myoclonus likely in the setting of chronic metabolic derangements.

Over the next six days of his hospitalization (day 37 to day 42), the patient had no improvement in his symptoms despite his up-titrated anti-seizure regimen. On day 42 of hospitalization, the patient was found to have severe vitamin B6 deficiency at a level of 1.8 µg/L (normal range of 3.4-65 µg/L). As a result, the patient was started on vitamin B6 supplementation via a loading dose of 100 mg per day for seven days (day 42 to day 48). Two days following the initiation of pyridoxine supplementation, the patient experienced complete resolution of his symptoms, which was sustained for the remainder of his hospitalization. He was discharged on a maintenance dose of 50 mg per day.

**Table 1:** Vitamin levels of the patient during hospital admission compared to normal reference ranges

Vitamin	Patient Value	Normal Range
Vitamin B6	1.8 μg/L	3.4-65 μg/L
Vitamin B9	15.1 ng/mL	>3 ng/mL
Vitamin B12	521 pg/mL	232-1245 pg/mL
Vitamin D, 25-Hydroxy	33.8 ng/mL	30-100 ng/mL

## Discussion

In the present case, the patient’s pyridoxine deficiency was likely multifactorial, as he had several risk factors including end-stage renal disease, malnutrition, and alcohol use disorder. Upon review, there was no offending medication contributing to his deficiency. However, this specific vitamin deficiency is uncommon in the United States due to fortification of foods such as cereals [7]. This case is relevant due to the lack of vitamin B6 testing in the initial workup for patients with neuropathy and other neurologic complaints. Pyridoxine deficiency may lead to a multitude of adverse effects, including peripheral neuropathy, stomatitis, depression, and neurologic disorders. Chronic alcoholism is a major cause of vitamin B6 deficiency and is found in the majority of patients with alcohol use disorder. Since the liver is the primary organ responsible for the metabolism of ethanol and is the site of PLP metabolism, the adverse effect of pyridoxine deficiency will occur due to acetaldehyde accelerating the degradation of intracellular PLP [8]. Additionally, a retrospective study in 2021 showed that vitamin B6 supplementation may reduce levetiracetam-associated irritability [9].

In our patient, there was a significant concern for potential breakthrough seizures, hence the modification of his levetiracetam doses. Our patient’s episodes of seizures one month prior to admission were likely a result of his pyridoxine deficiency, as alternative etiologies were ruled out with neuroimaging and laboratory studies. Alcohol withdrawal seizures were also ruled out due to his history of chronic alcoholism. He was initially placed on CIWA monitoring; however, no signs or symptoms of alcohol withdrawal were noted. As previously stated, pyridoxine deficiency may lead to neurologic disorders such as encephalopathy and seizures. Due to its important role in the synthesis of GABA, a deficient level of pyridoxine decreases the production of GABA and therefore lowers the seizure threshold. This patient had no clinical improvement on levetiracetam monotherapy until the discovery of his severe pyridoxine deficiency and subsequent supplementation. This suggests that our patient’s ongoing myoclonic jerks, tremors, and neuropathy were partially, if not completely, explained by his pyridoxine deficiency.

## Conclusions

This case demonstrates a rare cause of neurologic dysfunction, including myoclonic jerks, neuropathy, and tremors despite anti-seizure therapy. It is imperative to broaden the differential and consider pyridoxine deficiency in the workup of neurologic complaints, especially in patients with high-risk conditions such as malnutrition, renal failure, and alcohol use disorder.
